# ALD based nanostructured zinc oxide coated antiviral silk fabric†

**DOI:** 10.1039/d2ra02653h

**Published:** 2022-07-04

**Authors:** Udit Kumar, Candace R. Fox, Corbin Feit, Elayaraja Kolanthai, Jeremy Sheiber, Yifei Fu, Sushant Singh, Parag Banerjee, Griffith D. Parks, Sudipta Seal

**Affiliations:** Advanced Materials Processing and Analysis Center, Department of Materials Science and Engineering, University of Central Florida Engineering 1 Rm 207, 12800 Pegasus Dr Orlando FL 32816 USA Sudipta.seal@ucf.edu; Department of Materials Science and Engineering, University of Central Florida Orlando FL 32816 USA; Burnett School of Biomedical Sciences, College of Medicine, University of Central Florida Orlando FL 32827 USA; Amity Institute of Biotechnology, Amity University Chhattisgarh Raipur-493225 C.G India; NanoScience Technology Center (NSTC), University of Central Florida Orlando FL 32816 USA; Renewable Energy and Chemical Transformation (REACT) Faculty Cluster, University of Central Florida Orlando FL USA; Florida Solar Energy Center (FSEC), University of Central Florida Orlando FL USA; Biionix Cluster, College of Medicine, University of Central Florida Orlando FL 32816 USA

## Abstract

The COVID-19 pandemic has underscored the importance of research and development in maintaining public health. Facing unprecedented challenges, the scientific community developed antiviral drugs, virucides, and vaccines to combat the infection within the past two years. However, an ever-increasing list of highly infectious SARS-CoV-2 variants (gamma, delta, omicron, and now ba.2 stealth) has exacerbated the problem: again raising the issues of infection prevention strategies and the efficacy of personal protective equipment (PPE). Against this backdrop, we report an antimicrobial fabric for PPE applications. We have fabricated a nanofibrous silk-PEO material using electrospinning followed by zinc oxide thin film deposition by employing the atomic layer deposition technique. The composite fabric has shown 85% more antibacterial activity than the control fabric and was found to possess substantial superoxide dismutase–mimetic activity. The composite was further subjected to antiviral testing using two different respiratory tract viruses: coronavirus (OC43: enveloped) and rhinovirus (RV14: non-enveloped). We report a 95% reduction in infectious virus for both OC43 and RV14 from an initial load of ∼1 × 10^5^ (sample size: 6 mm dia. disk), after 1 h of white light illumination. Furthermore, with 2 h of illumination, ∼99% reduction in viral infectivity was observed for RV14. High activity in a relatively small area of fabric (3.5 × 10^3^ viral units per mm^2^) makes this antiviral fabric ideal for application in masks/PPE, with an enhanced ability to prevent antimicrobial infection overall.

## Abstract 2

This study presents an antiviral self-cleaning fabric for masks/PPE applications with protection against human coronavirus.

## Graphical abstract

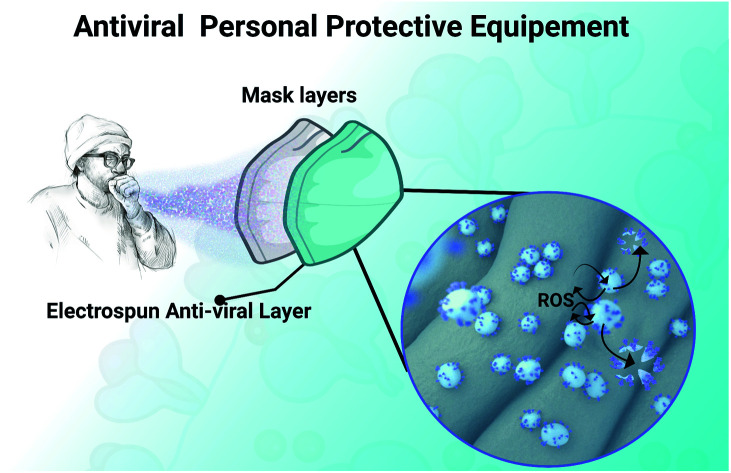

## 1. Introduction

We are in the midst of one of history's worst pandemics, and it has been more than two years since the 1^st^ outbreak was reported in Wuhan, China. More than 494 million cases of COVID-19 were reported, with more than 6.1 million deaths attributed to this highly contagious disease worldwide. More than 1 million deaths have been reported in the United States alone.^1^ The unprecedented nature of the challenge has kept the scientific community on tenterhooks. A great impetus has been given to research involving vaccine developments to drug design to formulate effective virucides.^2–6^ Despite the significant progress in this area, new variants of the SARS-CoV-2 virus (alpha, beta, gamma, delta, omicron, and now ba.2 stealth variant) have left us behind and necessitated further research. Particularly the highly communicable variants with breakthrough infections, despite vaccines, demonstrate the need for better infection prevention techniques. New variants of SARS-CoV-2 show higher infectivity and transmission.^7^

Antimicrobial fabrics for personal protective equipment could be one of the ways to mitigate the rapid spread of highly communicable variants of this virus. There could be various ways to achieve this goal. The most logical thought process demands the presence of active antiviral agents in the fabrics. One of the most common pathways antiviral agents act is by producing reactive oxidative species (ROS). Inorganic nanoparticles exhibit antioxidative behavior, such as cerium oxide nanoparticles^4,8–11^ and zinc oxide (ZnO) nanostructures.^12–16^ ZnO is an economical option for this application because it is not a rare earth oxide, which helps in cost and availability. As such, ZnO is the material we are investigating in the current work. Incorporating such antioxidative agents into the fabrics is an innovative approach to combat virus and bacteria spread. Desirable characteristics of this approach include; a minimal quantity of active materials required, antioxidative activities should not be impeded by architecture, and the highest surface-to-surface contact between active materials and pathogens should be ensured for maximum activity. As the antiviral properties of the ZnO are surface-dependent properties,^13^ coating a very thin layer of ZnO on top of the fabrics could be a feasible approach. One of the common and industrial techniques for precise coating of thin films is atomic layer deposition (ALD). For zinc oxide, ALD precursors are relatively simple and cheap. We have used the ALD technique to incorporate very thin ZnO layers into the fabrics. It illustrates a unique nanoarchitecture of active materials - fabrics for various applications needing flexible antioxidative fabrics.

**Fig. 1:**
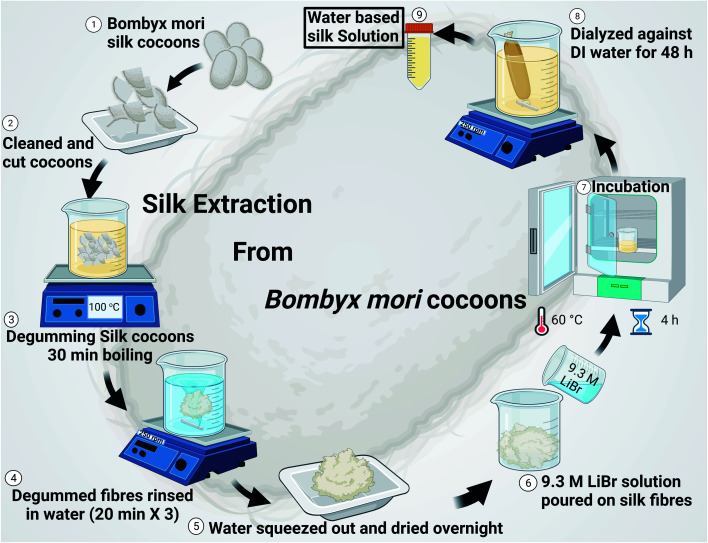
Schematic diagram shows silk fibroin extraction from *Bombyx mori* raw silk cocoons. It involved cleaning and degumming silk cocoons, followed by washing and drying. Dried fibroin was then digested in LiBr solution by incubating it at 60 °C. The solution obtained was then subjected to dialysis against DI water.

**Fig. 2:**
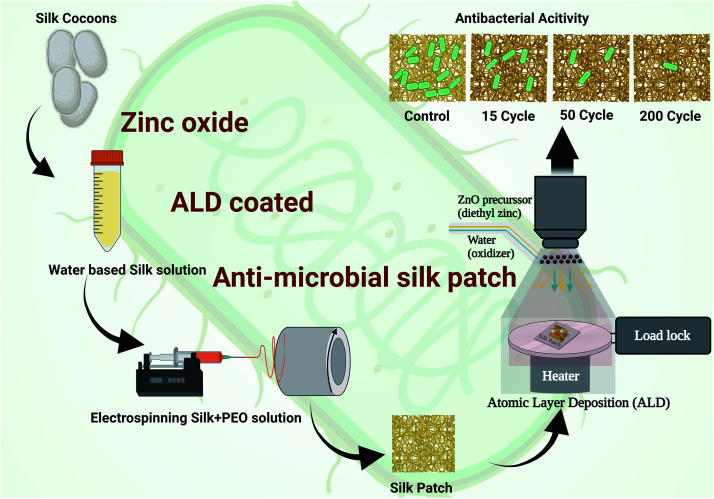
A schematic diagram shows experimental procedures and steps, silk extraction, electrospinning to get a patch followed by ZnO deposition using atomic layer deposition (ALD) followed by antibacterial activity testing.

Electrospun silk fibers have excellent biocompatibility^17^ and are easy to process in a laboratory setting, making them an ideal choice for the current investigation where we are interested in studying the antimicrobial properties of ZnO ALD thin films over fabrics. ZnO nanostructures/ALD films also have excellent biocompatibility, and it is a well-studied field.^18–20^ Additionally, silk has been utilized in personal protective equipment (PPE) as a protective barrier, especially in face coverings.^21^ We extracted silk fibers from *Bombyx mori* silk cocoons using a LiBr-based process (see Fig. 1), explained further in this report and our earlier works.^17^ Electrospun silk patches were then subjected to the ZnO ALD process for three different thicknesses, followed by antibacterial testing (see Fig. 2). Antioxidative properties were analyzed using a superoxide dismutase mimetic (SOD) mimetic assay. Samples with the best antibacterial and antioxidative properties were tested for antiviral characteristics.

As a major public health concern, human respiratory viruses impose a substantial burden on the economy and the health care industry. Every year, approximately 40 billion dollars in direct and indirect medical costs are associated with non-influenza-related respiratory virus infections in the United States alone.^22^ These illnesses are associated with non-enveloped rhinoviruses and enveloped coronaviruses, which continuously circulate in the human population and cause reoccurring seasonal infections. Common circulating coronaviruses, such as strain OC43, tend to start surging in the winter and are sustained into spring.^23^ Better infection prevention techniques are the need of the hour, and the materials we are developing in this work can help with that. Especially in relation to ongoing coronavirus (COVID-19) pandemics, there is a critical demand for better strategies, such as more effective PPE, to prevent transmission and diseases associated with respiratory viruses. Flexible antimicrobial fabrics can find application in PPE and as one of the layers in face masks, offering better protection.

## 2. Materials and methods

### 2.1 Silk extraction

The formation of silk fibroin solution and silk extraction from *Bombyx mori* silk cocoons was done according to the method described in our earlier published work.^17^ In brief, *Bombyx mori* silk cocoon technical grade was obtained from Aurora silk, USA. Every individual silk cocoon is cut into eight half inches size pieces (as shown in Fig. 1) and cleaned by hand. 5 g of cleaned and cut pieces were boiled in 2 L of 0.02 M Na_2_CO_3_ solution for degumming. Degummed silk was washed in DI water (3*x*) for 20 min. Each, and then water was squeezed out, followed by overnight drying at room temperature. Silk fibroins were obtained and then digested in 9.3 M LiBr solution (a ratio of 1 : 4 was maintained of the weight of silk fibroin in g: volume of 9.3 M LiBr solution in mL), which was incubated at 60 °C for 4 h in an oven, followed by dialysis against DI water for 48 h (4 water changes). After dialysis, the yellowish silk solution was centrifuged at 9000 rpm to remove any undigested silk fibroin impurities. The solution obtained was then stored in an airtight container in the fridge at 4 °C. The silk solution obtained is of ∼7% (wt vol^−1^) concentration in general.

### 2.2 Electrospinning

The silk electrospinning process followed in the current work is based on one of our earlier published work.^17^ In brief, 5% PEO (polyethylene oxide) stock solution was prepared and stored in the fridge. A 5 : 1 (vol/vol) ratio mixture of silk solution and 5% PEO solution was prepared in a vial by subjecting it to mild stirring for 15 min. The homogeneous solution was then drawn using a 5 mL syringe, and a 23G needle was then mounted on the syringe pump (as shown in Fig. 2). A potentiostat was used to create a 20 kV (current 2 A) potential difference between the needle on the syringe containing silk-PEO solution and collector drum rotation at 2000 rpm. Positive voltage lead was connected to the needle, and ground lead was connected to the collector drum. A flow rate of 1 mL h^−1^ was set in the syringe pump. The electrospinning process was carried out until we got the silk patch's desired thickness (∼0.004 mm).

### 2.3 Atomic layer deposition (ALD)

Atomic layer deposition (ALD) was done using a similar process described in our earlier reported work.^24^ In brief, ALD deposition of ZnO was done using a Veeco FIJI Gen 2 ALD system. DEZ (diethyl zinc) was used as a ZnO precursor (DEZ ≥ 52 wt% in hexane, Sigma Aldrich). DI water was used as the co-reactant (Sigma Aldrich). The precursor and oxidizer pulses were 0.06 s long and separated by 6 s of Ar gas purge. A low deposition temperature of 100 °C was used to prevent silk patches from any degradation. Every ALD batch was run with a silicon chip standard subjected to *in situ* spectroscopic ellipsometry (SE) to measure the silicon equivalent thickness of the ZnO layer. J.A. Woollam® M-2000 equipment was used to perform *in situ* SE measurement of film thickness deposited by ALD, at an incident angle of 69.5°, with a 190 to 1690 nm wavelength range. The COMPLETE EASE software was used to build the optical models for thin film analysis consisting of a Si substrate with a native oxide of ∼1.7 nm of SiO_2_, followed by a ZnO (GenOsc), given in the materials library, for the zinc oxide top-layer, respectively. Full details on the optical parameters used in the GenOsc model are given in the ESI (S1. Ellipsometry†).

### 2.4 Characterizations

Surface morphologies and elemental analysis of electrons pinned nanofibrous silk patches were done using scanning electron microscopy (FEG SEM Zeiss ULTRA-55) equipped with NORAN System 7 EDS system for elemental detection. X-ray photoelectron spectroscopy was performed using a Thermo fisher ESCALAB-250Xi spectrometer using Al-Kα as the source. Experiments were performed in an ultra-vacuum (<6 × 10^−9^ mbar). XPS data analysis and peak deconvolution were done using Thermo Avantage software. The Characterization techniques followed to analyze silk patches are similar to our earlier published report.^17^ TEM sample preparation for virus was done using phosphotungstic acid (99.9% pure, sigma Aldrich) as a negative strain. The staining procedure followed in this word was based on an earlier published report by Gelderbloom *et al.*^25^ TEM sample preparation for fibers was done after calcination of ZnO–Silk sample, followed by dispersion in ethanol and drop-casting on a copper TEM grid. TEM imaging was performed using FEI Tecnai F30 TEM and JEOL TEM-1011.

### 2.5 Antibiofilm testing

Biofilm assay was performed on pure and different cycle ZnO coated (2–45 nm thickness) silk fiber mat. 6 mm diameter silk fiber mat was taken for testing in 96 well plates. Initially, three different log numbers of *E. coli* were added to the testing plate containing pure and ZnO coated silk fiber mat and incubated for 48 h at 37 °C. After incubation, the excess media was removed from each well, and the biofilm formed over the mat surface was dried for 30 min. At 37 °C. To each well, 200 µL of 0.001% safranin (Sigma Aldrich, Dye content ≥80%, quality level 200) was added and incubated for 20 min at room temperature. Excess safranin was removed and washed with PBS twice, and stained biofilm was left to dry for 30 min at 37 °C. The biofilm is dissolved in an acetone/ethanol mix (80 : 20 v/v). The plate was read in a 96-well plate reader for absorbance at 490 nm. The experiment was performed with six replicates for each condition, and the results were averaged. Experiments were performed under ambient white light present in a lab (standard fluorescent lighting).

### 2.6 Antioxidative properties measurement

Antioxidative properties were analyzed using a SOD mimetic assay. A SOD assay kit (Dojindo SOD Assay Kit-WST, #S311-10) was used as per the instructions provided by the manufacturer. 6 mm diameter disks of silk patches were used as samples; absorbance was recorded for 20 min in a UV/Vis spectrometer (PerkinElmer). Blank solution without any sample was used as a negative control, and aq ceria nanoparticles were used as a positive control. Aq ceria nanoparticles were reported to have high SOD activity,^8,26^ and it has been used as a control in our earlier works.^11^

### 2.7 Antiviral testing

Antiviral testing was done using a procedure similar to our earlier published report with minor changes,^4^ detailed procedure is described in this section. HCT-8 cells were cultured in 10% heat-inactivated fetal calf serum (HI FBS, Gibco, Thermo Fisher Scientific) Roswell Park Memorial Institute medium (RPMI 1640, Gibco, Thermo Fisher Scientific). RD and HeLa cells were cultured in 10% HI FBS Dulbecco modified Eagle medium (DMEM, Gibco, Thermo Fisher Scientific). Human coronavirus OC43 (ATCC, catalog number VR-1558) was grown at 33 °C in HCT-8 cells. Media from virus-infected cells was clarified by centrifugation, and aliquots were quickly frozen and stored in the −80 °C freezer. OC43 stocks titers were determined using a standard 50% Tissue Culture Infectious Dose assay (TCID_50_) on 96-well plates (Falcon, Thermo Fisher Scientific) using confluent RD cells. Briefly, solutions were serially diluted in DMEM containing 0.38% Bovine Serum Albumin (BSA) as a carrier protein. Cells were washed with PBS and incubated with diluted virus solutions for 1 h at 33 °C. Cells were washed with PBS and replaced with 2% HI FBS DMEM, followed by a 4 days (d) incubation at 33 °C. Cells were then washed with PBS and stained with a crystal violet solution as described previously.^4^ TCID_50_ values were calculated by the Spearman & Kärber algorithm as previously described.^27^ Human rhinovirus 14 (RV14, ATCC, catalog number VR-284) was grown at 33 °C in HeLa cells. 3 d post-infection (DPI), attached cells and dislodged cells in media were combined by centrifugation, followed by three rounds of quick freeze and thaw. Stocks were then clarified by centrifugation, and aliquots were quickly frozen and stored at −80 °C. RV14 stock titers were quantified by a TCID_50_ assay in 96-well plates using confluent HeLa cells.

Approximately 1 × 10^5^ TCID_50_ units of each respective virus were delivered to either uncoated or 45.3 nm (200 ALD cycles) ZnO-coated silk 6 mm discs in 96-well plates. Titers of virus recovered as initial input infectivity was determined at time zero. Samples were exposed to a LED light (Ustellar 60 W LED work light) at a distance of 5 inches from the plates. At the incubation times indicated in the figures, media and silk discs were vortexed and washed in an assay tube containing 0.5 mL DMEM plus BSA as a carrier protein. Samples were then analyzed for infectivity according to respective virus quantification assays as described above. Values are the mean of three independent samples, and the standard deviation is represented by error bars.

### 2.8 Statistical analysis

Statistical analysis for anti-biofilm studies was done using Origin software. For antiviral studies, statistical analysis was performed using GraphPad unpaired student's *t*-test, with * indicating *p*-value < 0.05 and ** indicating *p*-value < 0.01.

## 3. Results and discussions

### 3.1 Characterizations

**Fig. 3:**
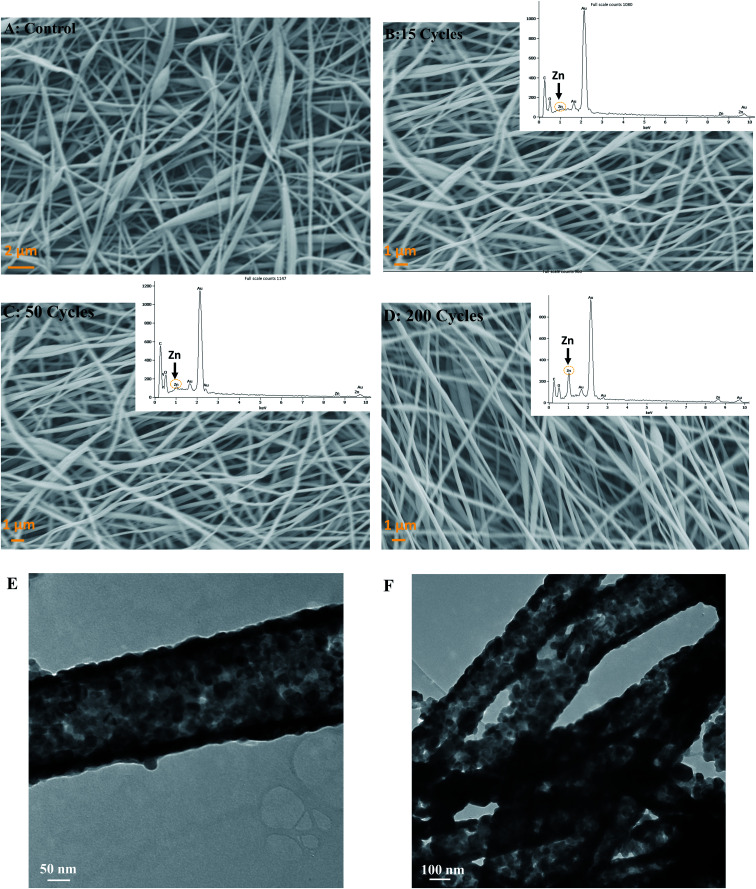
Shows the SEM-EDS analysis of the control and ZnO ALD coated silk patches. (A) shows the control silk patch with no ZnO ALD. (B) shows the one with 15 cycles (2.42 nm) of ZnO ALD, (C) shows the one with 50 cycles (8.48 nm) of ALD done, and (D) has 200 cycles (45.3 nm) of ZnO ALD done on top. EDS graphs with elemental analysis are shown in an inset for (B, C, and D). It is evident 200 cycles ZnO ALD sample (D) has the most prominent Zn peak. (E and F) show the TEM images of the 200 cycles (45.3 nm) ZnO ALD sample after calcination at 600 °C for 1 h, burning up and removing silk fibers and leaving behind a ZnO layer on fibers. TEM image shows ZnO were coated uniformly on the silk fibers.

All ALD experiments were conducted with a silicon standard due to the inability to monitor ZnO thickness directly on silk *via* SE. Therefore, a silicon wafer was present alongside silk patches in the ALD chamber and used as a thickness reference. For 15, 50, and 200 cycles of ALD deposited at 100 °C, the thickness of ZnO was found to be 2.42 ± 0.015 nm, 8.48 ± 0.024 nm, and 45.30 nm ± 0.007 nm, respectively (For more details, please see ESI S1: ellipsometry†). The average growth rate of ZnO on SiO_2_ was 1.8 ± 0.36 Å per cy, which is consistent with previous reports of ZnO ALD.^28^ All the samples were analyzed using FE-SEM and EDS (energy dispersive spectroscopy) to study surface topology, fibers composition, and elemental analysis. Silk fibers were ∼140 ± 45 nm in thickness (Thickness estimation was done using ImageJ software). Silk fibers varied in thickness, mostly thin (around 100 nm), but some had higher thickness values. SEM image for control, 15, 50, and 200 cycles (2–45 nm thickness) ZnO ALD is shown in Fig. 3A–D, respectively. EDS results are also shown in the inset for ZnO-coated samples. It is evident Zn peak is more prominent in the 200 (45.3 nm) ZnO ALD cycle sample.

Fig. 3E and F show TEM images of 200 cycles (45.3 nm) ZnO ALD sample after calcination at 600 °C for 1 h. Calcination was done to burn off the silk and leave behind the ZnO layer. As we can see, ZnO layers are in fiber shape, confirming a uniform deposition on silk fibers. It is also indicative of unique ZnO-fiber architecture, which can ensure high surface-to-surface contact between antimicrobial agent ZnO and any microbes on top of the fabric.

**Fig. 4:**
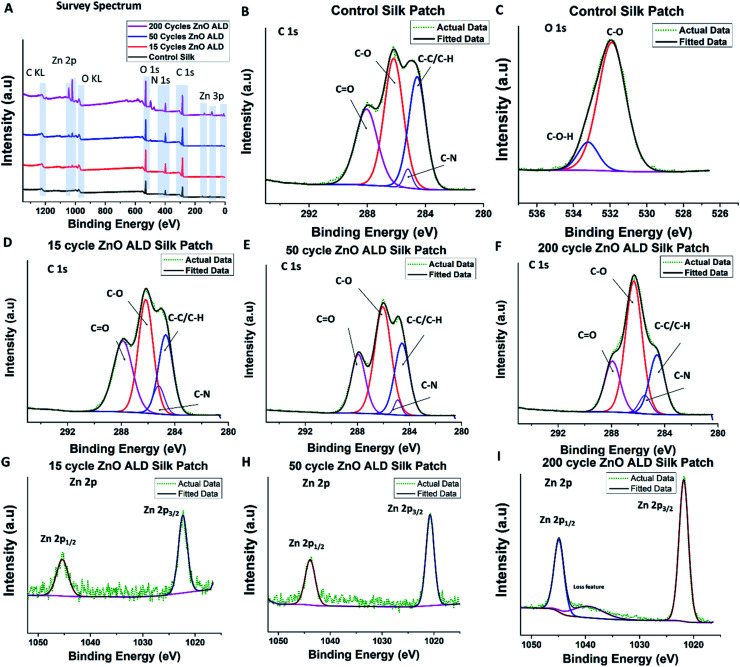
XPS analysis was performed on the control silk patch, and ZnO ALD coated silk patches. (A) show a Y offset stacked XPS survey spectrum for control silk patch, 15, 50, and 200 ZnO ALD cycle silk patches. (B and C) shows C 1s and O 1s spectrum for control silk patches. (D, E, and F) shows C 1s of 15 (2.42 nm), 50 (8.48 nm), and 200 (45.3 nm) ZnO ALD cycle silk patches. Similarly, (G, H, and I) show Zn 2p spectrum of 15 (2.42 nm), 50 (8.48 nm) and 200 (45.3 nm)ZnO ALD cycle silk patches. As per our expectation we have observed C, Zn, O and N atoms in XPS survey spectrum. In C1s scan we have observed peaks corresponding to C–C/C–H, C–N, C–O, and C=O interactions, also marked on (B, D, E, and F). There is an apparent trend in increase and decrease in intensity of these peaks, which has been summarized in Table 1. In Zn2p scans, apart from doublet peaks of Zn^2+^/zinc-oxide Zn 2p1/2 peak and Zn 2p3/2 peak, loss feature was also observed in 200 ZnO (45.3 nm) ALD cycle silk sample (I).

X-ray photoelectron spectroscopy can be a valuable tool for studying the chemical bond structure of the electrospun silk patch. Although despite its usefulness, reports with meaningful XPS analysis of electrospun silk fibers remain sparse.^17,29,30^ We have performed XPS analysis on control and ZnO ALD coated silk patches. From the survey spectrum (shown in Fig. 4A), C, O, and N were present in all samples. In addition, the Zn atom was also present in ZnO-coated silk patches. The atomic percentage of Zn and O estimated based on the XPS survey spectrum is shown in Table 1 Zn at% increased with the number of ZnO ALD deposition cycles performed on the patches. It again confirms the deposition of ZnO on top of silk patches supporting our observation with EDS-SEM. Furthermore, we have performed different elemental scans, C 1s, O 1s, and Zn 2p. As silk is a carbon-based natural polymer, the C 1s scan provides us with useful insights.

**Table 1:** Chemical composition (at.%) electrospun silk patches control, ZnO ALD coated samples and C 1s peak area percentage for carbon components identified

Samples	C 1s – peak area% (Int. Peak *AR*_i_ = *A*_i_/Σ*A*)				Atomic%	
	C–C/C=C	C–O	C=O	C–N	Zn	O
Control silk patch	0.31	0.39	0.27	0.02	0	14.07
15 Cycles ZnO ALD (2.42 nm)	0.27	0.39	0.27	0.07	0.48	22
50 Cycles ZnO ALD (8.48 nm)	0.27	0.47	0.22	0.02	1.04	23
200 Cycles ZnO ALD (45.3 nm)	0.22	0.53	0.19	0.05	6.07	25.14

We have observed C–C/C–H bonds, C–N bonds, C–O bonds, and C=O bonds in line *Bombyx mori* silk-PEO fibers (extracted in a similar manner) structure.^17^ Silk from *Bombyx mori* generally consists of repeating sequences of six amino acid/residues (Gly-Ala-Gly-Ala-Gly-Ser),^31^ which explains the presence of different functional groups associated with amino acids C–N, C–O, and C=O. C–C/C–H is present due to aliphatic carbon chains present in the backbone chain and amino acids. C 1s scan plot control silk patch is shown in Fig. 4 B, for 15 ZnO ALD cycle (2.42 nm) in D, 50 ZnO ALD cycle (8.48 nm) in E similarly for 200 cycles (45.3 nm) in F. Taking C–C/C–H peak to be at 284.6 eV as standard accordingly peak position shifted. The peak for C–N was centered at 285.2 ± 0.23 eV, C–O peaked at 286.2 ± 0.12 eV, and the peak for C=O was centered at 287.9 ± 0.03 eV [for more details, please see ESI: S2.† XPS (X-ray photoelectron spectroscopy)]. Integrated peak area ratios were calculated for different identified peaks in the C 1s scan (int. peak *AR*_i_ = *A*_i_/Σ*A*). The area ratios are shown in Table 1. It is interesting to note that C–C/C=C *AR* (area ratio) decreases marginally with an increase in the number of ALD cycles (increase in ZnO thickness)., C=O *AR* also decreases marginally, but C–O *AR* increases significantly with the number of ALD cycles. This observation explains the nature of the process done on top of those silk patches, which is an ALD process. It involves exposing silk patches to zinc precursor and water vapor (act as oxidizer) at 100 °C in a high vacuum atmosphere. Exposure of silk to water vapor is very important and has been studied before.^32^ It is known to induce transformation to β sheets dominated structure. Although the goal of the ALD process was not to induce structure transformation, and it was not exposed to water for long as water pulses in the ALD recipe are of short durations, it does explain the change in chemical nature we are witnessing here. C–N AR was less significant and was not found to follow a trend. O1s scan was also performed, scan plot for control silk patch as shown in Fig. 4C.

We have also performed Zn 2p scans for ZnO ALD coated samples, Zn 2p1/2 peak was found to be centered around 1045.2 ± 0.24 eV, and Zn 2p3/2 peak was found to be centered around 1022.1 ± 0.24 eV, which is in line with reported XPS results for ZnO ALD thin films,^33^ it also confirms the presence of zinc oxide. Zn 2p scan plots for 15 (2.42 nm), 50 (8.48 nm), and 200 (45.3 nm) ZnO ALD cycles are shown in Fig. 4G–I. Interestingly, we have observed a loss feature hump/peak in Zn2p XPS scan of 200 ZnO ALD (45.3 nm) cycles sample.

### 3.2 Antibiofilm properties

**Fig. 5:**
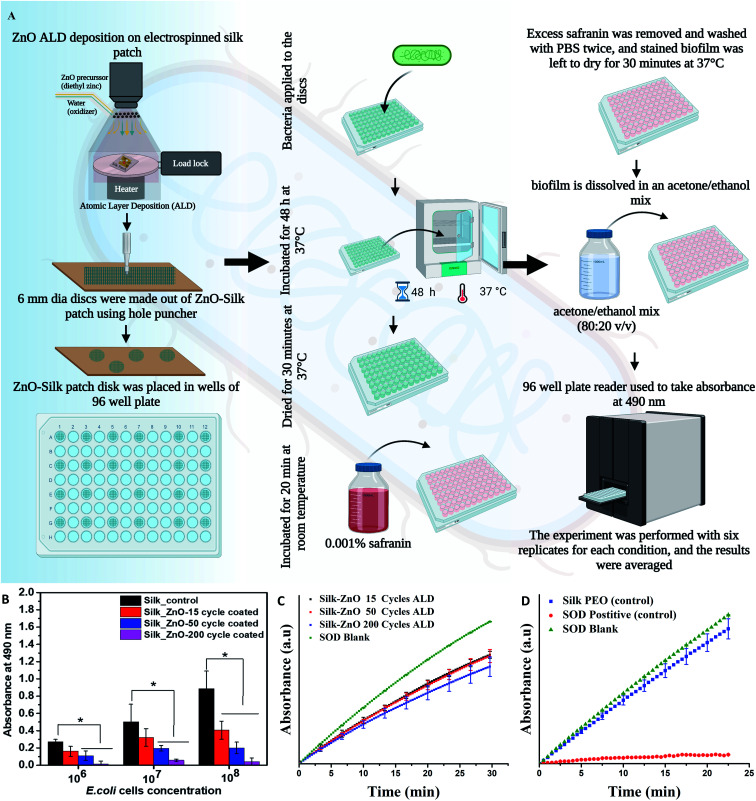
A) shows a schematic diagram of antibiofilm assay procedures, it was performed over pure and ZnO coated silk fiber mat using safranin. Initially, three different log values of *E. coli* bacteria were added on the control sample of silk fiber and test sample of different thickness ZnO coated silk fiber mat, analyzed after 48 h incubation at 37 °C. (B) shows results that were performed over pure and ZnO coated silk fiber mat using safranin. Three different log values of *E. coli* bacteria were tested (as shown on the *X* axis). In the highest concentration tested, a reduction of ∼85% was observed compared to the control. Antibiofilm was evaluated based on six replicate samples with standard deviation (*n* = 6, *p* < 0.05). * Indicate significant difference when compared to control sample of silk fiber mate. (C and D) show SOD assay results. It was conducted to estimate the SOD mimetic activity of the ZnO ALD coated silk patches. (B) 6 mm disk of 15 (2.42 nm), 50, and 200 (45.3 nm) ALD cycle samples checked for SOD, (C) control silk patch with respect to a positive control of aq ceria nanoparticles.

We have investigated the antibiofilm efficacy of ZnO-coated silk fiber mat using *E. coli* strain. A schematic diagram of the process is shown in Fig. 5A. The biomass of *E. coli* cells was quantitatively analyzed, and it has shown significant inhibition of biofilm formation when increasing the thickness of the ZnO layer on silk fiber mat by ALD technique when compared to the uncoated silk fiber mat. The results show that the highest thickness coated ZnO 200 cycles of ALD (45.3 nm thickness) silk fiber mat exhibited significant anti-biofilm activity with a reduction of ∼85% when compared to the control (shown in Fig. 5B). The uncoated fiber mat showed an increase in the bacterial biomass with respect to the increase in the number of *E. coli* inoculated, indicating that the uncoated fiber mat here has no antibiofilm activity. Existing studies have stated that ZnO has the potential to inhibit enzyme activity, disintegrate the bacterial cell membrane, inhibit DNA synthesis, interrupt energy transduction, damage genomic DNA, mitochondrial damage, intracellular outflow, apoptosis, and mitotic arrest. These properties of the ZnO could have caused bacterial death, thereby inhibiting the biofilm formation over the ZnO-coated mat surface area.^34^

In most of the studies *E. coli* has been chosen as a model organism because among the nosocomially caused infection *E. coli* plays a vital role and cause respiratory illness, including ventilator-associated pneumonia (VAP). *E. coli* is easily transmittable in between patients–patients in hospital environment. In the respiratory secretions of cystic fibrosis (CF) patients, *E. coli* has been isolated frequently.^35^ Hence to evaluate the antibacterial efficiency of the ZnO-Silk sample *E. coli* has been preferred as a study organism. *E. coli* has also been tested with different ZnO nanostructures.^36–39^ A preliminary experiment with ZnO helped in understanding the antimicrobial potential of the material. Based on our findings we procced for the antiviral testing experiments.

### 3.3 SOD mimetic properties

SOD mimetic properties of the ZnO ALD coated silk samples were estimated using a SOD mimetic assay. SOD activity plots are shown in Fig. 5C and D. 200 cycles ZnO ALD (45.3 nm) coated silk patch was found to be 31% SOD active compared to the SOD blank. We have also seen improvement in SOD percentage activity with an increase in thickness of the ALD ZnO layer (see Fig. 5C). In the control experiment, the control silk-PEO patch was found to be 10% SOD active, and positive control aq ceria nanoparticles were found to be 95% active compared to SOD blank (see Fig. 5D). SOD mimetic assay shows a moderate activity for ZnO ALD coated silk patch. Further studies are desired in this direction, and it is part of our future work.

SOD mimetic activity of ZnO nanostructures has been studied before, and it is attributed to two different mechanisms.^13,34,40^ Zinc oxide is a semiconductor when illuminated by visible or UV light. It can lead to electron–hole pair generation due to the excitation of an electron from appropriate band/energy levels. When interacting with moisture/water, these electrons lead to ROS generation.^41^ ROS generation in the dark or not in the presence of an excitation source majorly is attributed to defect structure which can be engineered and controlled by nanoarchitecture. Singly ionized oxygen vacancies which are present on the surface of ZnO nanostructures can interact with the atmosphere/moisture/water to form superoxide radical.^12^ It is explained in detail in a later section. All the SOD mimetic assays were performed in the absence of light, as light may interfere with the reaction.

### 3.4 Antiviral properties

**Fig. 6:**
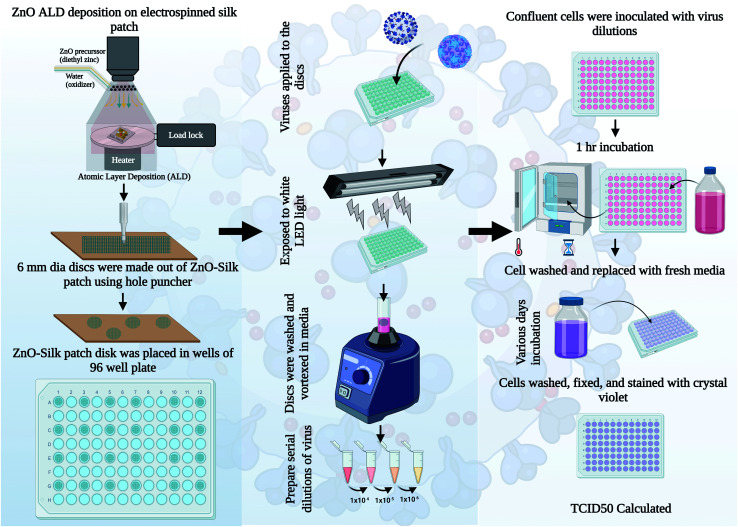
Schematic representation of the ZnO ALD coated silk disc antiviral experiments. ZnO silk patches were prepared. Approximately 1 × 10^5^ TCID_50_ units of respective virus was delivered to each disc. Input virus infectivity was measured at time zero on untreated silk discs. Discs were exposed to a LED light. At indicated incubation times, discs were vortexed in media, washed, and diluted. Samples were then analyzed for infectivity according to respective virus quantification assays.

After studying different ALD ZnO cycles coated silk patches for antibacterial and antioxidative properties, we observed silk-ZnO 200 cycles ALD sample has the best performance. For our further antiviral studies, we have only tested silk-ZnO 200 cycles ALD samples (ALD, 45.3 nm). Inactivation of human viruses associated with upper respiratory tract infections was investigated as described in Fig. 6. Uncoated or (ALD, 45.3 nm) 200 cycles ZnO coated silk patch discs were prepared, and approximately 10^5^ TCID_50_ units of either coronavirus OC43 or rhinovirus RV14 were applied to the discs. Discs were exposed to a LED light source for indicated times, followed by samples analyzed for infectious virus quantification.

**Fig. 7:**
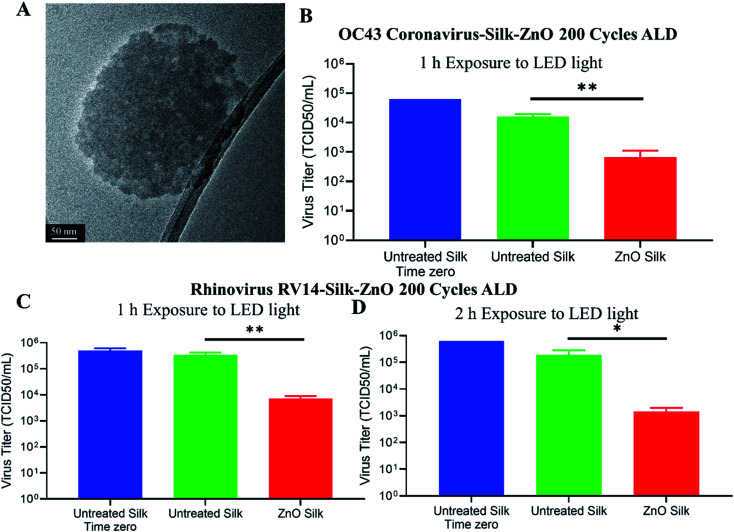
200 cycle ZnO coated silk discs inactivated rhinovirus and coronavirus OC43. A shows HR-TEM image of deactivated coronavirus OC43. Approximately 1 × 10^5^ TCID_50_ units of coronavirus OC43 (B) and rhinovirus (C and D) were applied to 200 cycle ZnO coated silk 6 mm discs in a 96-well plate. Input virus was quantified at time zero on untreated silk, as shown in blue bars. After 1 h (B and C) or 2 h (D) LED light exposure, silk discs and media were analyzed for remaining infectious virus according to the respective virus quantification assays. Values are the mean of three independent samples and the standard deviations are represented by error bars. * Indicates *p*-value < 0.05 and ** indicates *p*-value < 0.01 comparing light exposed untreated silk *versus* ZnO coated silk discs.

We next sought to determine the extent to which ZnO-coated silk patches could inactivate human coronavirus OC43. A TEM image of deactivated OC43 coronavirus is shown in Fig. 7A. Roughly 10^5^ infectious TCID_50_ units of OC43 virus were applied to uncoated or ZnO-coated silk discs (Fig. 7B). Time zero input virus was quantified as 10^5^ TCID_50_ per mL. Silk discs were then exposed to a LED light source for 1 h, followed by processing for the remaining infectious virus. About 10^4^ TCID_50_ per mL of infectious OC43 was recovered from the exposed untreated silk patches. Strikingly, ZnO-coated silk discs had reduced titers to 6 × 10^2^ TCID_50_ per mL, a 95.8% loss in infectious OC43 as compared to the control after 1 h exposure to LED light.

To determine the extent to which ZnO-coated silk patches could inactivate human rhinovirus RV14, about 10^5^ infectious TCID_50_ units of RV14 virus were applied to uncoated or ZnO-coated silk discs. Time zero infectivity was determined as 5 × 10^5^ TCID_50_ per mL input virus. Silk discs were then exposed to a LED light source for 1 h, followed by a media wash, and samples were processed for remaining infectious viruses. About 1 × 10^5^ TCID_50_ per mL of infectious RV14 was recovered from uncoated exposed discs, whereas the ZnO-coated silk discs had reduced titers to 7 × 10^3^ TCID_50_ per mL (Fig. 7C). These results demonstrated that light-exposed ZnO silk discs inactivated over 97% of infectious rhinovirus in only one hour. We next wanted to investigate the potency of ZnO-coated silk to inactivate RV14 with a longer light exposure incubation. Approximately 10^5^ infectious TCID_50_ units of RV14 virus were distributed to uncoated or ZnO-coated silk discs (Fig. 7D). Silk discs were then exposed to a LED light source for 2 h, followed by a media wash, and processed for the remaining infectious virus. After 2 h of light exposure, untreated silk maintained 5 × 10^5^ TCID_50_ units per mL of infectious RV14, while ZnO silk discs had a 2-log reduction in infectious RV14 titers, a 99% reduction as compared to controls. Taken together, these results demonstrated the potency of ZnO-coated silk material to inactivate over 95% of both rhinovirus and coronavirus in just 1 h.

**Fig. 8:**
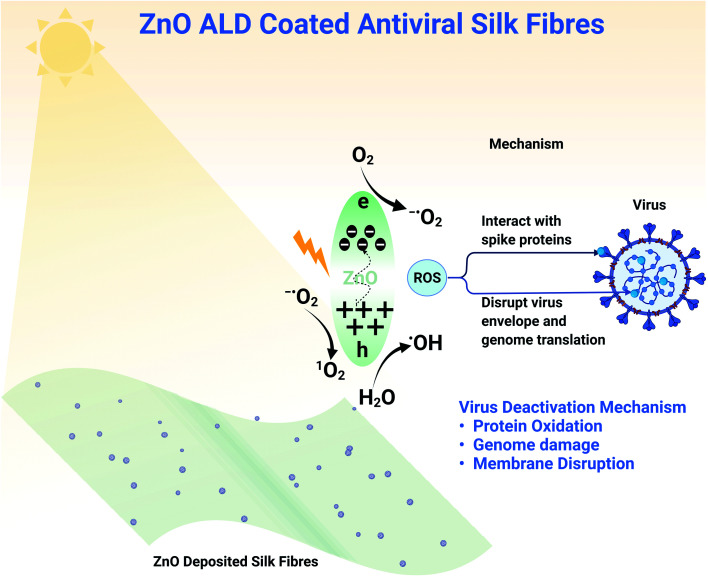
Schematic diagram showing viral deactivation mechanism of ZnO ALD coated silk patch. Zinc oxide nanostructure with a bandgap in between 3.10 to 3.37 eV is a semiconductor. White light illumination to ZnO nanostructures generated electron–hole pairs, which interact with surroundings to generate ROS species. ROS species interact with viruses. Depending on the conditions, ROS can cause protein oxidation, RNA damage, and membrane disruption, ultimately causing virus deactivation.

Zinc oxide nanostructures have a bandgap of between 3.10 to 3.37 eV, making them a semiconductor and enabling photocatalytic activity. Upon excitation with visible light, there is the momentary generation of electron–hole pair. When reacted with surrounding moisture/water/oxygen, these electron–hole pairs lead to the formation of ROS species (shown in a schematic diagram Fig. 8).^12,41–43^ Electron hole pair generation depends on the bandgap and the excitation source (white LED light here), but the generation of ROS species depends on electron transfer between ZnO and the surrounding. The transfer of electrons can be better understood by the redox potentials of these reactions and energy positions of the valance band (VB) and conduction band (CB) of ZnO. O_2(aq)_/^−^˙O_2_ reaction has −0.16 eV, ^1^O_2_/O_2_ reaction has 1.88 eV and H_2_O/˙OH reaction as 2.2 eV redox potentials. −0.31 and 2.89 eV are the energy positions of CB and VB of ZnO in absolute. Now comparing redox potentials and absolute energy positions, the formation of ROS species such as ^1^O_2_ (singlet oxygen), ˙OH (hydroxyl ion), ^−^O_2_ (superoxide ions) seems feasible. In our work, we have deposited ZnO on top of a silk patch using ALD. When exposed to light, zinc oxide nanostructures produce these ROS species that interact with different virus components (shown in a schematic diagram Fig. 8). ZnO nanoparticles have been reported to be genotoxic^40^ due to oxidative stress. The main mechanisms behind viral deactivation are likely; interaction with viral attachment proteins, for example spike protein, protein oxidation, genome damage, and membrane disruption, all of which have been attributed to ROS.^44^ Effective deactivation mechanisms add support to our claim of developing antiviral fabrics for application in PPE. With white light illumination (abundantly available), antiviral fabrics can be utilized in masks/PPE. However, the ZnO nanostructure produces ROS in the dark, although less than what it produces under illumination. We haven't observed much decrease in viral counts in our experiments for experiments performed in the absence of LED light (for one h).

It has been more than two years since the beginning of pandemic, and masks wearing have been hailed as effective way to fight this airborne disease.^45^ New variants of SARS-CoV-2 (alpha, beta, gamma, delta, omicron, and now ba.2 stealth variant) has been reported to have some degree of vaccine resistance with high infectivity and transmission.^7^ Studies about different kind of masks and their effectiveness against SARS-CoV-2 came forward.^46^ It brought the focus back on infection prevention methods and effectivity of masks. For infection-prone environments, guidelines were updated to use appropriate respirators^47^ for adequate protection. High infectivity and transmission necessitated the development of antiviral masks.^46^ Our work also aims to present a concept that can be used for those applications. We have used an industrially ubiquitous technique to develop an antiviral fabric. Even though we have used silk patches due to their high biocompatibility,^17^ our work could also be emulated for other fabrics, which is part of our future work. We have presented a proof of concept of ZnO deposited feasible antiviral fabrics with potential application as masks layer or other PPEs.

## 4. Conclusions

The current study presents a unique architecture of flexible antiviral fabric that ensures high surface-to-surface contact. Atomic layer deposition technique was utilized to coat ZnO on electrospun silk fabric and further characterized using SEM, TEM, XPS, with ZnO film thickness monitored using *in situ* SE. A 2–45 nm (silicon equivalent) thickness ZnO coated silk patch was further studied for SOD mimetic and antibacterial activity using the *E. coli* bacterial strain. Silk patch with 45.3 nm ZnO coating has shown the best activity, 31% SOD active, and reduction of ∼85% biofilm *versus* control. 45.3 nm ZnO coated sample was further subjected to antiviral testing against human viruses associated with human upper respiratory tract infections following exposure to white LED light; namely, enveloped OC43 coronavirus and non-enveloped RV14 rhinovirus. We observed more than 95% reduction (from ∼1 × 10^5^ initial counts) in viral counts for both OC43 coronavirus and rhinovirus within 1 h of illumination for 6 mm 45.3 nm ZnO coated silk sample discs. Rhinovirus was further tested under 2 h of illumination, with a 99% reduction in viral counts observed. This study serves as a proof of concept for an antiviral fabric with antiviral activities against respiratory tract infections with potential application in masks/PPE.

## Author contributions

Conceptualization: UK, SS, EK, S. Seal; investigation: UK, CRF, CF, EK, JS, YF; formal analysis: UK, CRF, CF, EK, JS, YF; supervision: SS, GP, PB, S. Seal; visualization and writing: UK, CRF, CF, EK, YF; editing: UK, CRF, CF, EK, JS, YF, SS, PB, GP, S. Seal.

## Conflicts of interest

There are no conflicts to declare.

## Note added after first publication

This article replaces the version published on 4th July 2022, which contained errors in the Abstract text.

## Supplementary Material

RA-012-D2RA02653H-s001
