# Supplementary material for: ALD based nanostructured zinc oxide coated antiviral silk fabric
Source: RSC Adv. 2022 Jul 4;12(30):19327–39. doi: 10.1039/d2ra02653h (PMC9251734; doi:10.1039/d2ra02653h)
Supplement: RA-012-D2RA02653H-s001 [file RA-012-D2RA02653H-s001.pdf]

Electronic Supplementary Material (ESI) for RSC Advances.
This journal is © The Royal Society of Chemistry 2022

Supporting Information: ALD based Nanostructured Zinc Oxide coated Antiviral Silk Fabric

*Udit Kumar[1,2], Candace R. Fox[3], Corbin Feit[2], Elayaraja Kolanthai[1,2], Jeremy Sheiber[3], Yifei Fu[1,2], Sushant Singh[4], Parag Banerjee[2,5,6,7], Griffith D. Parks[2], Sudipta Seal[1,2,5,8]**

[1]Advanced Materials Processing and Analysis Center, Department of Materials Science and Engineering, University of Central Florida, Orlando, FL, 32816, United States of America.

[2]Department of Materials Science and Engineering, University of Central Florida, Orlando, FL, 32816, United States of America.

[3]Burnett School of Biomedical Sciences, College of Medicine, University of Central Florida, Orlando, FL, 32827, United States of America.

[4]Amity Institute of Biotechnology, Raipur-493225, C.G, India

[5]NanoScience Technology Center (NSTC), University of Central Florida, Orlando, FL, 32816, United States of America.

[6]Renewable Energy and Chemical Transformation (REACT) Faculty Cluster, University of Central Florida, Orlando, FL
[7]Florida Solar Energy Center (FSEC), University of Central Florida, Orlando, FL

[8]Biionix Cluster, College of Medicine, University of Central Florida, Orlando, FL, 32816, United States of America.

*Corresponding author: Sudipta.seal@ucf.edu; Engineering 1 Rm 207, 12800 Pegasus Dr, Orlando, FL 32816, USA

S1. Ellipsometry

Sample: POST 200 cy ZnO at 100C on Si-native

Fit Results	Optical Model
MSE = 4.043<br>ZnO Thickness = 45.30 ± 0.006 nm	- Layer # 2 = ZnO (GenOsc) Thickness # 2 = **45.30 nm** (fit)<br>**Show Dialog**<br>- **e1 Components**<br>Einf = 1.826<br>UV Pole Amp. = 40.2335 UV Pole En. = 6.750<br>IR Pole Amp. = 0.2271<br>- **e2 Components**<br>Oscillator Menu: **Add** **Delete** **Delete All** **Sort**<br>Fit Menu: All None **Amp.** **Br.** **En.**<br>1: Type = PSemi-M0 Amp1 = 4.154 Br1 = 0.0897<br>Eo1 = 3.324 WR1 = 1.7753 PR1 = 0.938<br>AR1 = 0.437 O2R1 = 0.645<br>2: Type = Gaussian Amp2 = 1.580523 Br2 = 1.8737 En2 = 4.961<br>3: Type = Gaussian Amp3 = 1.900752 Br3 = 4.3791E-05 En3 = 3.374<br>Layer # 1 = INTR_JAW Thickness # 1 = 1.57 nm<br>Substrate = SI_JAW

Experimental and Model Generated Data Fits

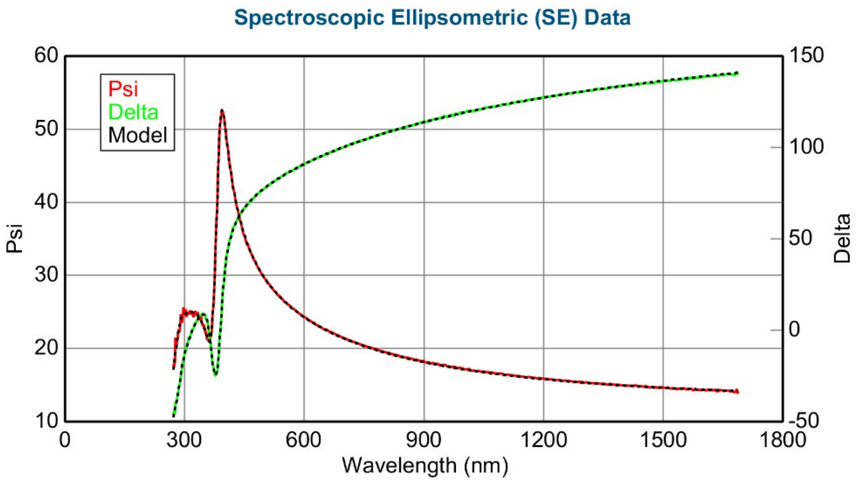

Sample: in situ 50 cy ZnO at 100C on Si-native

Fit Results	Optical Model
MSE = 9.102<br>Thickness # 2 = 8.34 ± 0.025 nm<br>ZnO Thickness = 8.34 ± 0.025 nm	- Layer # 2 = ZnO (GenOsc) Thickness # 2 = **8.34 nm** (fit)<br>**Show Dialog**<br>- **e1 Components**<br>Einf = 1.923<br>UV Pole Amp. = 39.4332 UV Pole En. = 7.999<br>IR Pole Amp. = 0.4196<br>- **e2 Components**<br>Oscillator Menu: **Add** **Delete** **Delete All** **Sort**<br>Fit Menu: All None Amp. Br. En.<br>1: Type = PSemi-M0 Amp1 = 3.857 Br1 = 0.1561<br>Eo1 = 3.521 WR1 = 2.0746 PR1 = 0.785<br>AR1 = 0.331 O2R1 = -0.00040140<br>2: Type = Gaussian Amp2 = 2.631504 Br2 = 2.6711 En2 = 6.559<br>3: Type = Gaussian Amp3 = 0.388221 Br3 = 0.7921 En3 = 0.220<br>Layer # 1 = INTR_JAW Thickness # 1 = 1.70 nm<br>Substrate = SI_JAW

Experimental and Model Generated Data Fits

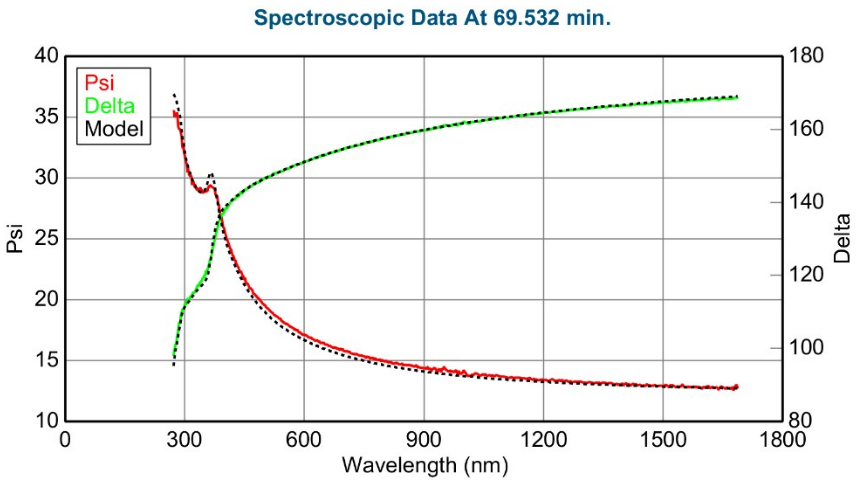

Sample: in situ 15 cy ZnO at 100C on Si-native

Fit Results	Optical Model
MSE = 5.265<br>Thickness # 2 = 0.01 ± 0.014 nm<br>Angle Offset = 0.0847 ± 0.007324	- Layer # 2 = ZnO (GenOsc) Thickness # 2 = **2.42 nm** (fit)<br>**Show Dialog**<br>- **e1 Components**<br>Einf = 1.923<br>UV Pole Amp. = 39.4332 UV Pole En. = 7.999<br>IR Pole Amp. = 0.4196<br>- **e2 Components**<br>Oscillator Menu: **Add** **Delete** **Delete All** **Sort**<br>Fit Menu: All None Amp. Br. En.<br>1: Type = PSemi-M0 Amp1 = 3.857 Br1 = 0.1561<br>Eo1 = 3.521 WR1 = 2.0746 PR1 = 0.785<br>AR1 = 0.331 O2R1 = -0.00040140<br>2: Type = Gaussian Amp2 = 2.631504 Br2 = 2.6711 En2 = 6.559<br>3: Type = Gaussian Amp3 = 0.388221 Br3 = 0.7921 En3 = 0.220<br>Layer # 1 = NTVE_JAW Native Oxide = 1.71 nm<br>Substrate = SI_JAW

Experimental and Model Generated Data Fits

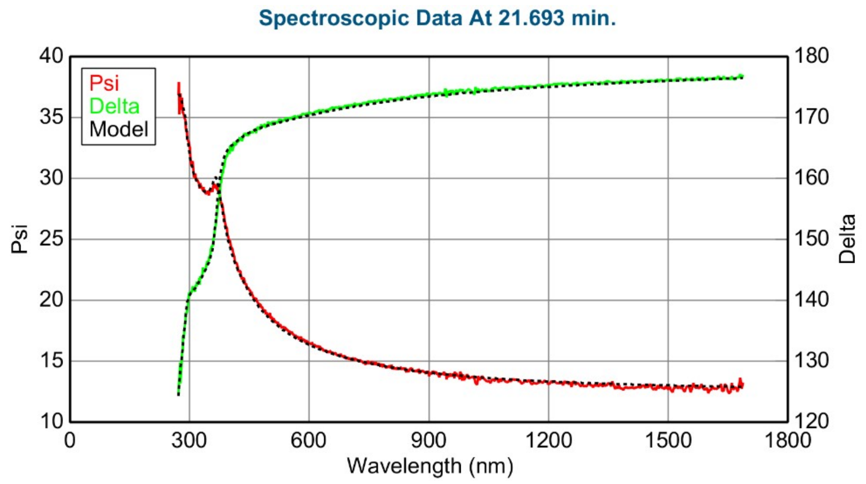

S2. XPS (X-ray photoelectron spectroscopy)

Sample Details	Peak Positions (eV)				Peak shift (eV)
	C-C/C-H	C-N	C-O	C=O	
Control silk	282.976	283.634	284.6	286.4	1.624
15 Cycle	283.048	283.691	284.59	286.41	1.552
50 Cycle	283.07	283.413	284.548	286.454	1.53
200 Cycle	283.17	284.077	284.9	286.519	1.43

Sample Details	Peak Positions			
	C-C/C-H	C-N	C-O	C=O
Control silk	284.6	285.258	286.224	288.024
15 Cycle	284.6	285.243	286.142	287.962
50 Cycle	284.6	284.943	286.078	287.984
200 Cycle	284.6	285.507	286.33	287.949
